# Design of a broadly reactive Lyme disease vaccine

**DOI:** 10.1038/s41541-020-0183-8

**Published:** 2020-05-01

**Authors:** Heather D. Kamp, Kurt A. Swanson, Ronnie R. Wei, Pradeep K. Dhal, Ram Dharanipragada, Aurelie Kern, Bijaya Sharma, Radek Sima, Ondrej Hajdusek, Linden T. Hu, Chih-Jen Wei, Gary J. Nabel

**Affiliations:** 1 Sanofi, 640 Memorial Dr, Cambridge, MA 01239 USA; 2 Department of Molecular Biology and Microbiology, Tufts University, 136 Harrison Ave, Boston, MA 02111 USA; 3 Institute of Parasitology, Biology Centre of the Czech Academy of Sciences, Ceske Budejovice, Czech Republic

**Keywords:** Immunology, Infectious diseases, Vaccines

## Abstract

A growing global health concern, Lyme disease has become the most common tick-borne disease in the United States and Europe. Caused by the bacterial spirochete *Borrelia burgdorferi* sensu lato (sl), this disease can be debilitating if not treated promptly. Because diagnosis is challenging, prevention remains a priority; however, a previously licensed vaccine is no longer available to the public. Here, we designed a six component vaccine that elicits antibody (Ab) responses against all *Borrelia* strains that commonly cause Lyme disease in humans. The outer surface protein A (OspA) of *Borrelia* was fused to a bacterial ferritin to generate self-assembling nanoparticles. OspA-ferritin nanoparticles elicited durable high titer Ab responses to the seven major serotypes in mice and non-human primates at titers higher than a previously licensed vaccine. This response was durable in rhesus macaques for more than 6 months. Vaccination with adjuvanted OspA-ferritin nanoparticles stimulated protective immunity from both *B. burgdorferi* and *B. afzelii* infection in a tick-fed murine challenge model. This multivalent Lyme vaccine offers the potential to limit the spread of Lyme disease.

## Introduction

Lyme borreliosis is a zoonotic disease caused by *Borrelia burgdorferi* sl and is transmitted to humans and canines by the bite of an infected tick. Four *Borrelia* species are responsible for the majority of infections globally. In the United States, *B. burgdorferi* is the most common cause of disease, while *B. afzelii*, *B. garinii*, and *B. bavariensis* cause disease in Europe, Asia and elsewhere. Lyme disease affects more than 300,000 people in the U.S. yearly, causing recurrent fatigue, cardiac arrhythmias, arthralgias, and neurological abnormalities^1,2^, costing the U.S. health care system more than $1.3 billion dollars a year^3^. In Europe, Lyme disease has spread to new regions geographically and its incidence has increased, estimated at 230,000 cases per year^4–7^. The CDC^8^ reports that up to 30% of Lyme disease patients do not display the characteristic rash, making diagnosis and treatment difficult. Additionally, serological tests, culture, and PCR are often negative early in Lyme disease^9^. The bacteria can spread from the skin to the heart, joints, and nervous system in individuals not treated with antibiotics early in infection. Although the majority of patients do well with antibiotic therapy, up to 20% of patients will continue to have symptoms after completion of treatment, sometimes lasting over 12 months^10^. Vaccination therefore represents a key public health intervention that would prevent Lyme disease by preventing primary infection. Essential to successful prophylaxis, a vaccine needs to protect both children and adults.

OspA is a lipoprotein expressed on the *B*. *burgdorferi* sl outer membrane surface when the bacteria reside in the tick gut. Antibodies against OspA, acquired with the blood meal as the tick feeds on vaccinated hosts, bind and kill the organism in the tick midgut before transmission can take place^11^. OspA-specific antibodies therefore prevent the transmission of *B. burgdorferi* from the tick vector to the human host. The previously licensed vaccine, LYMErix^TM^ validated the use of OspA as an immunogen against human Lyme disease against one strain of *Borrelia*^12^. Four other studies describe second generation OspA vaccines in mice in which the amino acid sequence 165–173 of *B. burgdorferi* OspA B31 was modified by site directed mutagenesis^13^ or replaced by the homologous sequence from non-arthritogenic *Borrelia* species^14–16^. In this study, we developed a nanoparticle-based Lyme vaccine by genetically fusing OspA to the N-terminus of *Helicobacter pylori* ferritin. Ferritin is a self-assembling nanoparticle composed of 24 subunits arranged in octahedral symmetry surrounding a hollow core^17^. It has been used successfully for presentation of antigens such as influenza hemagglutinin, Epstein-Barr virus gp350 and HIV envelope protein^18–20^. Here, we included OspA derived from seven of the major OspA serotypes found in strains worldwide in a prototype vaccine and tested its immunogenicity and efficacy in animal models.

## Results

### Design and characterization of OspA-ferritin nanoparticles

**Fig. 1:**
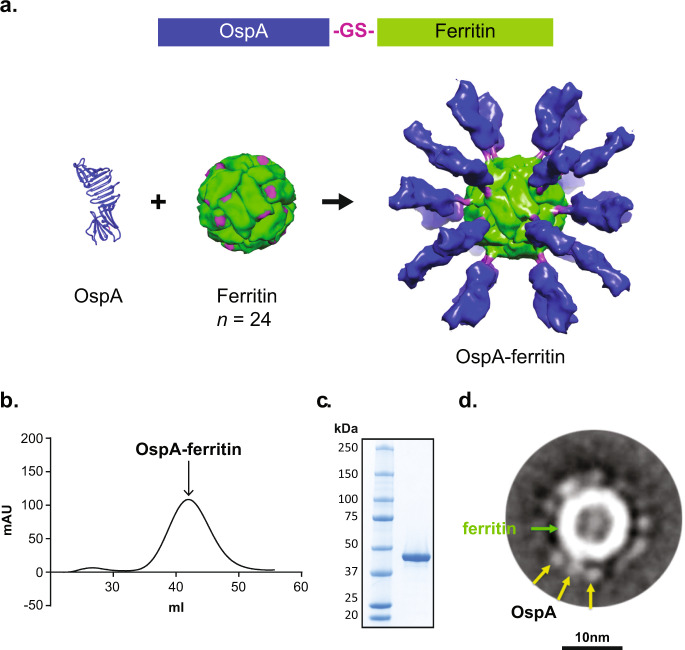
Design, expression and purification of OspA-ferritin nanoparticles. Schematic diagram of OspA fused to a modified *H. pylori* ferritin through a glycine–serine (GS) linker (A,top). Secondary structure of the transmembrane domain deletion of OspA, in which the carboxy-terminus of OspA where it is attached to ferritin is indicated (purple) (**a**, bottom left). The ferritin nanoparticle is composed of 24 monomers of *H. pylori* ferritin (**a**, bottom middle). The amino-terminal attachment site for OspA on ferritin is highlighted (purple). Structural model of the OspA-ferritin nanoparticle (**a**, bottom right). Ferritin (green), the GS linker (purple), and OspA (blue) is shown. A SEC profile of OspA-ferritin nanoparticle purification on a Superose 6 column (**b**). A SDS-PAGE gel of purified OspA-ferritin from *E.coli* (**c**). Annotated class averages of OspA-ferritin (318 particles) at 67,000x magnification (**d**). The ferritin cage appears as a strong circular density with a hollow center in the middle of the averages (green arrow). Each cage is surrounded by numerous, short, uniform spikes of OspA that appear circular or slightly oblong in shape (yellow).

We generated OspA-ferritin nanoparticles by genetically fusing unlipidated OspA to the amino-terminus of ferritin from *Helicobacter pylori* (Fig. 1a, top). OspA is a 31-kDa lipoprotein with an extended β-sheet structure composed of 21 consecutive antiparallel β-strands^21^ with only one carboxyl-terminal α-helix (Fig. 1a, bottom left). The 24 subunits of ferritin assemble spontaneously into a hollow spherical nanoparticle (Fig. 1a, bottom middle). The amino terminus of ferritin was designed to facilitate radial projection from the nanoparticle core^19,22^ that would optimize exposure of the 24 OspA proteins on the nanoparticle surface (Fig. 1a, bottom right).

OspA serotype 1 from *B. burgdorferi* strain B31 was fused to ferritin and expressed in *E. coli*. Nanoparticles of the expected size were isolated as measured by size exclusion chromatography (SEC) and dynamic light scattering (DLS). SEC revealed a single symmetrical peak (Fig. 1b), and DLS analysis documented the expected particle radius of 13 nm with low polydispersity (7.4%) (Supplementary data Fig. 1). OspA-ferritin expressed in *E. coli* migrated at a molecular weight of 47 kD (Fig. 1c). The majority of protective antibody epitopes on OspA are localized in the carboxy-terminal half of OspA due to the positioning of the protein in the outer membrane^23^. The known immunodominant protective antibody LA-2^24–27^, bound to OspA-ferritin nanoparticle at the same concentration as Recombitek^TM^ (Supplementary data Fig. 2), indicating that the protective epitopes in OspA are accessible when genetically fused to the ferritin nanoparticle.

Transmission electron microscopy negative staining and 2D class averaging analysis was performed on the OspA-ferritin nanoparticles (Fig. 1d). The ferritin cage appeared as a strong circular density with a hollow center in the middle. Each cage was surrounded by numerous, short, uniform spikes of OspA of oblong shape. The particles displayed an overall diameter ranging from ~194–220 Å, with a ferritin core diameter of 125 Å. Consistent with the proposed model, spikes extended uniformly in size, shape and orientation from the particle surface up to 45 Å in length. The OspA spikes were ~30 Å in width and tapered to minimal density at the glycine–serine linker to ferritin.

### Immunogenicity of serotype 1 OspA nanoparticles in mice and removal of LFA-1 homolog epitope

**Fig. 2:**
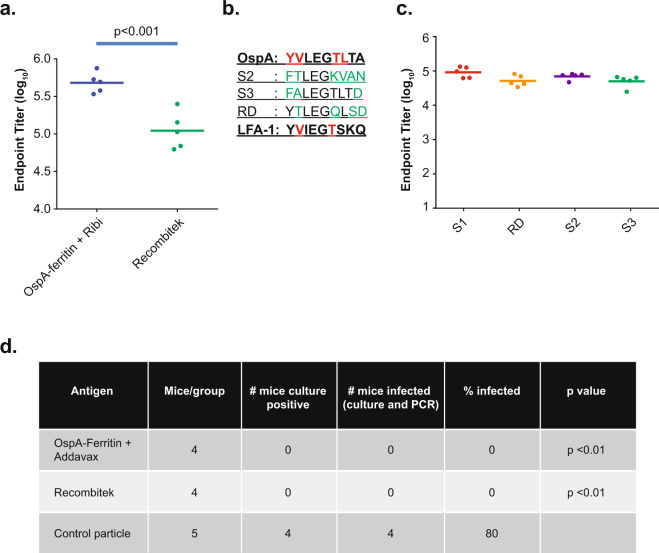
Immunogenicity and protection of serotype 1 OspA-ferritin nanoparticles. C3H mice were immunized intramuscularly with the molar equivalent of 1 µg of OspA-ferritin + Ribi adjuvant or 1 µg of Recombitek^TM^ at week 0 and week 4. ELISA titers were measured 2 weeks after the 2nd immunization. A student *t*-test was used to determine statistical significance. Antibody titer induced by OspA-ferritin vaccine is 4.4-fold higher than that of Recombitek with 24% coefficient of variation (CV) and *N* = 5 per group is powered (0.8 power) to detect a 3-fold difference (*p* < 0.001). The mean is indicated with a horizontal line. **a** Homology of human LFA-1 aa 326-173 to OspA Serotype 1 (S1) (aa165-173), to OspA Serotype 2 (S2), to OspA Serotype 3 (S3) and a rational design mutant of OspA (RD). **b** ELISA titers 2 weeks after the 2nd immunization of the indicated mutants. **c** C3H mice (*n* = 5) were immunized intramuscularly at weeks 0 and 4 with a molar equivalent of 1 µg dose of the indicated vaccines with the Addavax adjuvant. All vaccines were composed of OspA Serotype 1 with only the LFA-1 epitope replaced with either RD, S2, or S3 sequence. The ELISA plate was coated with OspA-His serotype 1. Protective efficacy of OspA-ferritin nanoparticle vaccine (**d**). Mice were vaccinated with a molar equivalent of 1 µg dose of antigens at week 0 and week 4. OspA-ferritin serotype 1 with the RD epitope was used. Mice were challenged with 5–6 ticks infected with *Borrelia burgdorferi* N40 strain (serotype 1) for 5 days two weeks after the second immunization and sacrificed two weeks later. Tissue samples from the heart, ankle and ear were cultured in BSK media with antibiotics for *B. burgdorferi* for 6 wks. Negative samples were tested by PCR for the presence of *B. burgdorferi*. All negative cultures were also PCR-negative.

To assess the immunogenicity of OspA-ferritin nanoparticles, C3H mice were immunized with adjuvanted serotype 1 OspA-ferritin nanoparticles or Recombitek^TM^, a canine vaccine containing full-length lipidated recombinant OspA from the same serotype. Recombitek^TM^ is composed of the same constituents as the previously licensed human vaccine LYMErix^TM^ ^12^, with the exception of the absence of adjuvant. Ab responses to these immunogens were determined by enzyme-linked immunosorbent assay (ELISA) to recombinant OspA. Immunization of mice with OspA-ferritin induced Ab endpoint titers 4.4-fold higher than Recombitek^TM^ two weeks after the boost (Fig. 2a; *p* < 0.001). The titer remained 4.4-fold higher than Recombitek^TM^ for an extended time frame (Supplementary data Fig. 3; week 25, *p* < 0.005).

One hypothetical concern about LYMErix^TM^ was the presence of an epitope with homology to the human leukocyte function-associated antigen-1 (LFA-1)^28^ (Supplementary data Fig. 4). Several second generation vaccines were developed after LYMErix^TM^ that removed this homologous epitope in OspA^13–16^. OspA serotype 1 is the only serotype that contains this sequence (Fig. 2b; YVLEGTLTA). Although the arithrogenic hypothesis was refuted^29^, we still chose to avoid this potential T-cell epitope and replaced it with either the OspA serotype 2 or 3 sequences, or introduced rationally-designed (RD) point mutations designed to eliminate it (Fig. 2b; S2, S3, or RD, respectively). For the RD epitope, we mutated surface-exposed amino acids predicted to eliminate homology to LFA-1 without destabilizing the β-sheet. When tested for their ability to elicit an Ab response in vivo, the immunogenicity of the mutant nanoparticles was comparable to the original OspA-ferritin nanoparticle (Fig. 2c). The serotype 1 RD mutant OspA-ferritin nanoparticle was used for all subsequent experiments.

### Protection against tick-borne challenge in vivo

To evaluate the protective efficacy of this prototype, we used a challenge model in which immunized or control mice were infected by ticks carrying *B. burgdorferi* serotype 1^30^. The serotype 1 RD OspA-ferritin nanoparticle was used to immunize mice. After immunization, mice were exposed to ticks infected with *B. burgdorferi* strain N40, sacrificed two weeks after the challenge, and tissues analyzed for the presence of *B. burgdorferi* by culture and PCR. Mice immunized with the OspA-ferritin vaccine in the presence of adjuvant showed no infection (0/4) as determined by cell culture or PCR in contrast to the negative control, ferritin, where four of five animals were infected (Fig. 2d; *p* < 0.01), thus documenting its protective efficacy in vivo. Mice immunized with Recombitek^TM^ as a positive control were protected (0/4) as expected, determined by bacterial culture and PCR. An antibody level of at least 100 ng/ml of LA-2 equivalent in human serum after vaccination with Lymerix has been shown to correlate with protection against serotype 1 *B. burgdorferi*^12^. It has also been reported that in mice the protective level of LA-2-like antibody is ≥320 ng/ml against serotype 1 challenge^31^. Our serotype 1 challenge studies clearly indicated that the serotype 1 OspA-ferritin vaccine elicited a protective titer of antibody and protected mice from tick challenge.

### A hexavalent vaccine is immunogenic and protective

**Fig. 3:**
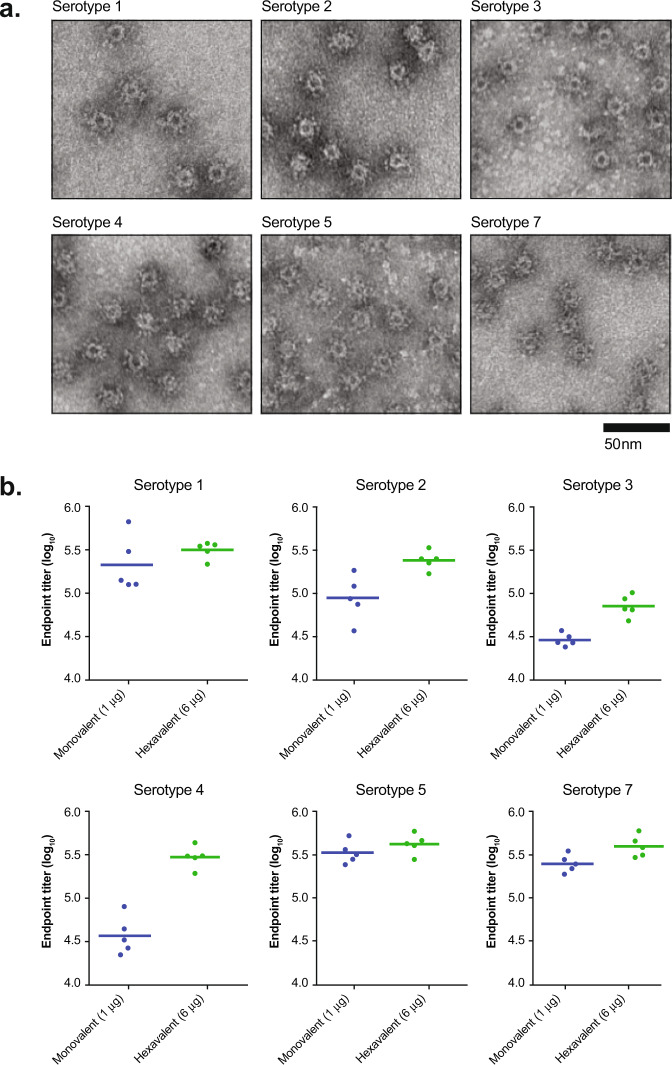
Immunogenicity of single and six component OspA-ferritin nanoparticles in mice. Transmission electron microscopy of OspA-ferritin serotypes 1–5, and 7 purified from *E. coli* (×68,000) (**a**). Immunogenicity of hexavalent vaccine in mice (**b**) C3H mice (*n* = 5) were immunized intramuscularly at weeks 0 and 4 with the indicated vaccine mixed with Alum and ELISA titers were analyzed 2 weeks later. The hexavalent vaccine included OspA from serotypes 1,2,3,4,5,7 at the molar equivalent of 1 µg each for a total of 6 µg, while the monovalent vaccine contained the molar equivalent of 1 µg of OspA plus 5 µg of empty ferritin particle. Empty ferritin alone is below the limit of detection. ELISA plates are coated with the specified serotype of OspA.

To generate a broadly cross-protective vaccine, we designed OspA-ferritin nanoparticles for serotypes 1, 2, 3, 4, 5, and 7. All OspA-ferritin fusion proteins, purified from *E.coli*, displayed the expected molecular weight of ~47 kDa (Supplementary data Fig. 5). Transmission electron microscopy confirmed the formation of all six OspA-ferritin nanoparticles (Fig. 3a). A six component vaccine composed of equimolar amounts of each OspA serotype was compared to a single serotype vaccine containing one OspA-ferritin serotype mixed with five molar equivalents of control ferritin. The six component vaccine induced a robust Ab response against all six OspA serotypes, and no competition was evident by mixing serotypes (Fig. 3b). In fact, the Ab responses typically increased with the hexavalent vaccine compared to the monovalent control (Fig. 3b, for example, note serotype 4).

**Fig. 4:**
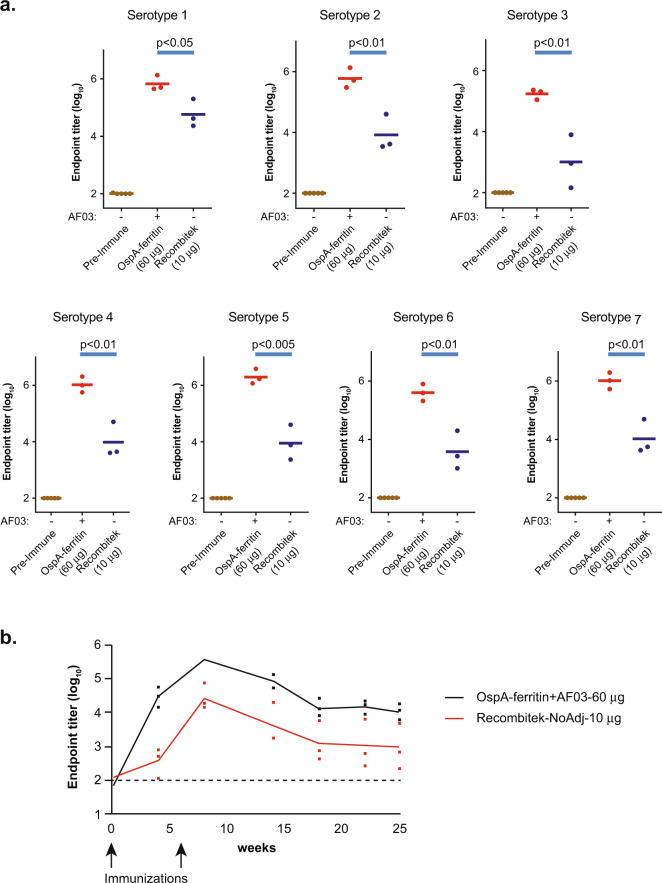
Immunogenicity of OspA-ferritin nanoparticles in rhesus monkeys. Immunogenicity of OspA-ferritin nanoparticle vaccines in rhesus monkeys (**a**). Rhesus monkeys (*n* = 3 per group) were immunized intramuscularly at week 0 and week 6 with the molar equivalent of 10 µg of each OspA-ferritin serotype (total 60 µg) with AF03 adjuvant and ELISA titers were analyzed 2 weeks later. Recombitek^TM^ was given at 10 µg dose. The ELISA plate was coated with the OspA serotype indicated in each panel. Time course of endpoint antibody titer in Rhesus monkeys (**b**). Indian rhesus macaques (*n* = 3 per group) were immunized intramuscularly at week 0 and week 6 with the molar equivalent of 10 µg of each OspA-ferritin serotype (total 60 µg) with AF03 adjuvant. Recombitek^TM^ was given at a 10 µg dose. The ELISA plate was coated with OspA serotype 1.

To model human immune responses to the hexavalent vaccine, it was further tested in non-human primates (NHP) with the AF03 adjuvant, which is a squalene oil-in-water emulsion adjuvant that has been approved for use in humans^32,33^. Again, the six component vaccine elicited an 11- to 200-fold higher Ab titer against all seven circulating *Borrelia* serotypes than Recombitek^TM^ (Fig. 4a). The Ab titer was sustained for at least 25 wks at levels significantly higher than Recombitek^TM^, demonstrating durability of the immune response (Fig. 4b).

**Fig. 5:**
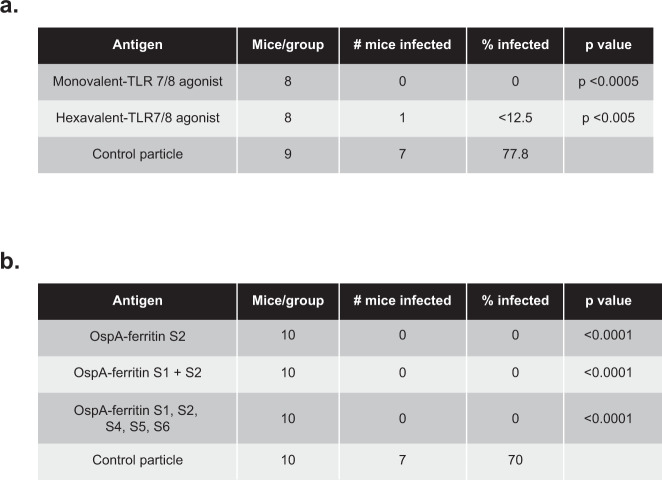
Protective efficacy of OspA-ferritin nanoparticle vaccines. Protective efficacy of hexavalent OspA-ferritin vaccine conjugated with TLR 7/8 agonist (**a**). Mice were vaccinated with the molar equivalent of 1 µg dose of antigen at week 0 and week 4. The monovalent vaccine contained 1 µg of OspA-ferritin serotype 1 conjugated to 3M-012. The hexavalent vaccine included OspA from serotypes 1–5, and 7 at 1 µg (6 µg total) each conjugated to 3M-012. Mice were challenged with 5–6 ticks infected with *Borrelia burgdorferi* N40 strain (OspA serotype 1) for 5 days two weeks after the second immunization and sacrificed 2 weeks later. Tissue samples from the heart, ankle and ear were cultured in BSK media with antibiotics for *B. burgdorferi* for 6 wks. Negative samples were tested by PCR for the presence of *B. burgdorferi*. A positive sample was positive either by culture or PCR. The difference in protection between monovalent (100%) and hexavalent (87.5%) was not statistically significant (*p* = 0.33). Protective efficacy of OspA-ferritin vaccine against *B. afzelii* (**b**). Mice were vaccinated with the molar equivalent of 1 µg dose of each antigen mixed with AF03 adjuvant at week 0 and week 4. Pentavalent vaccinated mice received the molar equivalent of 1 µg dose of S1, S2, S4, S5, and S6 (5 µg total). Mice were challenged with 5–6 ticks infected with *B. afzelii* CB43 strain (OspA serotype 2) for 5 days 2 weeks after the second immunization and sacrificed 2 weeks later. Tissue samples from the heart, bladder and ear were tested by qPCR for the presence of *B. afzelii*. A positive sample was positive in at least 2 tissues. A negative sample was negative in all tissues.

We also evaluated an alternative approach to enhancing the vaccine response by conjugating a Toll-like receptor TLR 7/8 agonist directly to ferritin using click chemistry (see Methods). This is a self-adjuvanting nanoparticle vaccine. The TLR 7/8 3M-012 agonist was coupled to the nanoparticle modified at serine 111 with cysteine using a two-step reaction of agonist linked to a polyethylene glycol (PEG) linker (Methods). Near complete conjugation of ferritin was observed, suggesting that each nanoparticle carried 24 molecules of agonist. The hexavalent vaccine conjugated to 3M-012 was tested in a tick-fed challenge model using *B. burgdorferi* strain N40 (Fig. 5a). Animals in the vaccinated groups, both monovalent and multivalent, showed substantial protection (100% and 87.5%, respectively) compared to the ferritin particle negative control, indicating that the TLR-conjugated vaccine was also highly protective against a live tick challenge. These results also demonstrate that multivalency does not affect potency of antibody response for protection. The ability to generate a multivalent vaccine without compromising the protective immune response is beneficial for broad protection from ticks carrying several different OspA serotypes, as typically found in European countries. Therefore, we have shown that the ferritin nanoparticle platform is protective against *Borrelia burgdorferi* with the use of different adjuvants and with multivalent vaccination combining several OspA serotypes (Figs. 2d, 5a).

To demonstrate protection against European strains of *Borrelia*, we tested our nanoparticle vaccine in a tick challenge model using serotype 2 *Borrelia* *afzelii*-infected ticks. Mice were immunized with nanoparticle vaccine of either serotype 2 alone, serotype 2 and serotype 1 together, or a pentavalent vaccine containing serotype 1, 2, 4, 5, and 6 (see Methods). While the mice that received the control particle were 70% infected in at least two tissues, the mice vaccinated with our nanoparticle vaccines were 100% protected with no positive tissues (Fig. 5b). Again, we also demonstrate that a multivalent vaccination does not affect the potency of protection against the serotype of interest (Fig. 5b).

## Discussion

An effective OspA vaccine requires the generation of antibodies with sufficient potency and durability to kill *B. burgdorferi* in the tick gut before transmission^11^. LYMErix^TM^, the previously licensed OspA vaccine, reduced serotype 1 infection by ~80% in vaccinated adults, and anti-OspA Ab levels were validated for serotype 1 as a correlate of protection in humans^12^. Our OspA-ferritin nanoparticle vaccine elicited markedly high Ab titers in mice and NHP, and the antibody titer is maintained at high levels for at least 6 months (Fig. 4, Supplementary data Fig. 3). Additionally, in both mice and NHP, we saw no adverse effects with OspA-ferritin nanoparticle vaccination indicating good tolerability (Supplementary data fig. 6).

Ferritin nanoparticle vaccines have been developed previously to improve the immunogenicity of challenging viral antigens, including influenza hemagglutinin, its highly conserved stem, HIV and EBV gp350^18–20,34^. Bacterial antigens have not typically been incorporated into the ferritin nanoparticle format. We show here that presentation of OspA antigen on a ferritin nanoparticle array improved its immunogenicity. The antigen-specific immune response could also be achieved by directly coupling a TLR ligand to acceptor sites engineered onto the surface of the ferritin core (Fig. 5a). Covalently coupling of TLR ligands to the nanoparticle ensures that the antigen and adjuvant are taken up by the same antigen presenting cells to enhance antigen presentation. During immunization, admixed low molecular weight adjuvants such as TLR ligands diffuse away quickly after injection, requiring larger doses for effective responses^35^. By targeted delivery of TLR ligands on a nanoparticle, the concentration of TLR agonist can be reduced yet stimulate an effective and specific Ab response that is protective (Fig. 5a).

More recently, a multivalent heterodimer protein subunit vaccine,VLA-15, was designed informatically and is now in phase II trials^15^. However, this antigen is a synthetic OspA fusion protein, which differs from OspA found on circulating strains. In contrast, the hexavalent vaccine described in this report matches native antigens on naturally occurring strains more closely and generates potent, synergistic immunity against OspA from all globally relevant strains. A non-lipidated recombinant OspA failed to elicit protective and durable Ab response again *B. burgdorferi* in mice^36^ and only partial protection in hamsters^37^. In contrast, our non-lipidated OspA is immunogenic and fully protective when displayed on a ferritin nanoparticle (Fig. 2a, d). Our OspA-ferritin nanoparticle vaccine also stimulated a higher antibody response than a full-length lipidated rOspA vaccine (Fig. 2a).

In both mice and NHP, we saw potent and durable antibody responses that are protective with our multivalent nanoparticle vaccine. Nanoparticle delivery of the OspA antigen is a novel vaccine approach to Lyme disease. We can display a validated antigen, OspA, in its natural conformation in an ordered multivalent array to improve immunogenicity. The platform is flexible and allows for the display of many different serotypes of OspA to protect against several strains of *Borrelia*. Because of its multivalent composition, by including *B. garinii, B. afzellii*, and *B. bavariensis* OspA subunits in our vaccine along with *B. burgdorferi*, the vaccine has the potential to protect against infection from U.S., European and Asian strains. The ability to express and purify the nanoparticle vaccine from *E. coli* is important because it can reduce manufacturing costs. We have also demonstrated that we can use multiple different adjuvants to achieve strong immunogenicity and protection. These features improve the safety, immunogenicity, and manufacturability of nanoparticle vaccines. Several previous vaccines have entered clinical trials based on mouse challenge data^15,38,39^. Taken together, this candidate OspA-ferritin nanoparticle vaccine has the potential to protect individuals against *Borrelia* infection worldwide and limit the spread of Lyme disease.

## Methods

### Strain and vector construction

Unplipidated OspA (deletion of first 25 aa) was synthesized from the following sequences: *Borrelia burgdorferi* strain B31(serotype 1) NBCI sequence ID WP_010890378.1, *Borrelia afzelii* strain PKO (serotype 2) NCBI sequence: WP_011703777.1, *Borrelia garinii* strain PBr (serotype 3) GenBank: CAA56549.1, *Borrelia bavariensis* (serotype 4) NCBI sequence WP_011187157.1, *Borrelia garinii* (serotype 5) GenBank CAA59727.1, *Borrelia garinii* (serotype 6) GenBank: CAA45010.1, *Borrelia garinii* (serotype 7) GenBank CAA56547.1.

The *H. pylori* ferritin with the 8 aa bullfrog sequence at the amino terminus was synthesized by Genescript^19^. OspA was fused to this modified ferritin with a GS linker. Three additional changes in the ferritin were incorporated to improve its functionality; N19Q, C31S, and S111C. The N19Q mutation removed a potential glycosylation site, while the S111C mutation introduced a cysteine residue on the surface that could be used to conjugate small molecules with click chemistry. Finally, we modified cysteine 31 to serine to minimize product heterogeneity due to cysteinylation at this site and to ensure that only one cysteine would be conjugated.

The pet21a vector was used to express both His-tagged OspA and OspA-ferritin nanoparticles in *E. coli*. The LFA-1 mutants in OspA serotype 1 were made by splicing by overlap extension (SOE) PCR^40^.

### Expression and purification

For purification of nanoparticles from *E. coli*, we used BL21 Star (DE3) (Invitrogen Cat #C601003). We induced the protein with 100 µM IPTG overnight at 16 °C. The cell pellet was lysed using sonication in Tris buffer pH 8, 50 mM NaCl. The filter sterilized supernatant was purified on an anion exchange column (HiTrap Q HP, GE), by collecting OspA-ferritin from the flow-through. The flow-through was then concentrated using an Amicon 100 MW cutoff filter (Millipore Cat #UFC910096) and nanoparticles were then further purified on a 120 ml Superose 6 preparatory SEC column in PBS pH 7.4. Endotoxin was removed by passing over a Sartobind Q column (Sartorius) and collecting the flow-through. We were able to express and purify Serotype 6 OspA-ferritin to add to our vaccine candidate using the same methods (Supplementary Fig. 7). The heptavalent vaccination gives a potent immune response against OspA serotype 6 similar to the monovalent vaccination (Supplementary Fig. 7C).

His_6_-tagged OspA (transmembrane domain deletions) of serotypes 1, 4, 5, 6, and 7 were purified from *E.coli* BL21 (DE3) (Invitrogen Cat #C600003), and serotype 2 and 3 were purified from Expi293F cells because expression in *E.coli* was poor. For *E. coli* purification, protein was induced at 500 µM IPTG for 5 h and cells were pelleted and frozen at −20 °C. The pellet was resuspended in 1% Triton in TBS buffer with Complete Protease Inhibitor (Sigma-Aldrich, Cat #11697498001) and sonicated to lyse cells. The supernatant was filter sterilized. For purification from mammalian cells, we used Expi293F cells transfected with plasmid DNA using FectoPRO transfection Reagent (Polyplus, Cat #116-100) per manufacturer’s instructions. Transfected cells were cultured on day 5 and the supernatant was collected and filtered. For both methods of expression, the remaining supernatant was run on a GE HiTrap HP 5-ml column (Cat #17-5248-02) attached to an AKTA Pure FPLC. The column was washed, loaded, and washed again with 20 mM imidazole in TBS. Final protein was eluted with 250 mM imidazole in TBS.

### Electron microscopy

OspA-ferritin nanoparticles were diluted 300-fold in TBS and imaged over a layer of continuous carbon supported by nitrocellulose on a 400-mesh copper grid. The grids were prepared by applying 3 µl of sample suspension to a cleaned grid, blotting away with filter paper, and immediately staining with uranyl formate. Electron microscopy was performed using an FEI Tecnai T12 electron microscope equipped with an FEI Eagle 4 k × 4 K CCD camera. High magnification images were acquired at normal magnification of 67,000 (0.16 nm/pixel). The images were acquired at a nominal underfocus of −1.9 µm to −0.8 µm and electron doses of ~30 e^−^/Å^2^. Individual particles in the 67,000x high magnification images were selected using automated picking protocols^41^. A reference-free alignment strategy was used based on the XMIPP processing package^42^. Algorithms in this package align the selected particles and sort them into self-similar groups or classes.

### Dynamic light scattering

Purified nanoparticles were loaded into a black 384-well plate with a clear bottom (Corning, Cat #3540) at a concentration ~0.4 µg/ml. Samples were read with a DynaPro plater Reader II (Wyatt) at a control temperature of 25 °C.

### *Ixodes scapularis* ticks

*Ixodes scapularis* tick larvae were obtained from National Tick Research and Education Center, Oklahoma State University (Stillwater, OK). *B. burgdorferi*-infected nymphs were generated using strain N40. Uninfected larvae were allowed to feed to repletion on *B. burgdorferi*-infected SCID mice. The engorged larvae were collected and allowed to molt into nymphs in 4–6 weeks at room temperature and high relative humidity. Prevalence of *B. burgdorferi* infection in fed larvae was determined by culture of a portion of the recovered ticks from each batch. Specifically, 10 ticks were selected for testing from each batch of ticks fed on mice. Ticks were washed with PBS, 3% H_2_O_2_ and then 70% ethanol sequentially. Ticks were dried and then placed in a tube containing BSK II supplemented with 50 µg/ml rifampicin, 100 µg/ml phosphomycin, 5 µg/ml amphotericin B. Ticks were crushed using a pestle and the tubes incubated at 35 °C. Cultures were monitored by darkfield microscopy for the presence of *B. burgdorferi*. The infection rate in ticks was calculated as the fraction of infected cultures over total.

### In vivo *Borrelia burgdorferi* challenge in mice

Tick challenge study was performed^30^. *Borrelia burgdorferi* challenge study experiments were performed following the guidelines of the American Veterinary Medical Association as well as the Guide for the Care and Use of Laboratory Animals of the National Institutes of Health. All procedures were performed with approval of the Tufts University Institutional Animal Care and Use Committee (Protocol# B2017-36). C3H/HeN mice were vaccinated intramuscularly with the indicated vaccines. OspA-ferritin nanoparticle was mixed with Addavax (Invivogen Cat#vac-adx-10) 1:1 where indicated. Recombitek^TM^ Lyme canine vaccine was obtained from Merial. Mice were vaccinated at week zero and week 4. At week 6, 2 weeks post-vaccination, mice were challenged by allowing 5 to 6 *B.* *burgdorferi*-infected nymphal ticks to feed to repletion. The fed nymphs were collected and assayed for *B. burgdorferi* infection by PCR. Two weeks after challenge, the mice were sacrificed and assayed for *B. burgdorferi* infection by culture of the ear, ankle and heart culture. Presence of *B. burgdorferi* was determined by observing the cultures by darkfield microscopy. Negative tissue samples were also tested by PCR specific to *B. burgdorferi*. A mouse was defined as infected with *B. burgdorferi* if one or more cultures were found positive by darkfield microscopy or by PCR.

### *Ixodes ricinus* ticks

*Ixodes ricinus* tick larvae were obtained from the breeding facility of the Institute of Parasitology, Biology Centre, Czech Academy of Sciences. *B.* afzelii-infected nymphs were generated using strain CB43^43,44^. Uninfected larvae fed to repletion on *B. afzelii*-infected C3H/HeN mice. The engorged larvae were collected and molted into nymphs in 4–6 weeks. Nymphs were considered infected if >90% were PCR positive.

### In vivo *Borrelia afzelii* challenge in mice

C3H/HeN mice were vaccinated intramuscularly with the indicated vaccines. OspA-ferritin nanoparticle was mixed with a squalene-based adjuvant, AF03 1:1 where indicated. Mice were vaccinated at week zero and week 4. At week 6, 2 weeks post-vaccination, mice were challenged by allowing 5 to 6 *B.* *afzelii*-infected nymphal ticks to feed to repletion. The number and weights of collected nymphs were recorded. Two weeks after challenge, the mice were sacrificed and assayed for *B. afzelii* infection by qPCR of the ear, heart, and bladder tissues. A mouse was defined as infected with *B. afzelii* if one or more cultures were found positive by qPCR. In most cases, positive mice were positive in 2–3 tissues. Negative mice were negative in all three tissues by qPCR. All experimental animals in the *B. afzelii* challenge study were treated in accordance with the Animal Protection Law of the Czech Republic No. 246/1992 Sb., ethics approval No. 161/2011. The animal experimental protocol was approved by the Czech Academy of Sciences Animal Care & Use Committee (Protocol Permit Number: 102/2016).

### Mouse vaccination

Animal experiments were carried out in accordance with all federal regulations and were approved by Sanofi Institutional Animal Care and Use Committee. C3H/HeN mice were vaccinated intramuscularly at week zero and week 4. ELISAs were run on serum from 2 wks post 2nd dose. Addavax (Invivogen Cat#vac-adx-10) was added in equal volume to antigen prior to immunization (1:1). Alum (Alyhydrogel ’85 2%; Brenntag – Cat# 21645-51-2) was added in equal volume to antigen prior to immunization (1:1). Ribi (Sigma adjuvant system Cat #S6322-1vl) was resuspended in 1 ml of PBS and vortexed for 1 minute and then added in equal volume to antigen prior to immunization (1:1). Recombitek^TM^ Lyme canine vaccine was obtained from Merial. No weight loss was observed in any immunized animals (Supplementary data Fig. 6).

### Rhesus monkey vaccination

The fifteen male and female rhesus macaques (*Macaca mulatta*), ages 2–9 years, were housed at BIOQUAL, Inc., in accordance with the recommendations of the Association for Assessment and Accreditation of Laboratory Animal Care International Standards and with the recommendations in the Guide for the Care and Use of Laboratory Animals of the United States—National Institutes of Health. The Institutional Animal Use and Care Committee of BIOQUAL approved these experiments IACUC protocol # 17-089 P. BIOQUAL’s animal facilities meet or exceed all AAALAC, USDA and OLAW standards for animal housing, and the surrounding environment. The vaccine candidates were delivered via the intramuscular route to the left and right quadriceps muscle. The animals were anesthetized prior to the immunizations by IM injection of Ketamine HCl (10-25 mg/kg). AF03 is a MF59-like adjuvant made in-house and was mixed in equal volume to the antigen just prior to injection. No adverse reactions noted in any immunized animals.

### Conjugation

Material was reduced to remove cysteinylation with 10 mM TCEP (Amresco K831-10G) in 50 mM Tris pH 8.5 for 1 h. The protein was then dialyzed into 100 mM Tris pH 8, 50 mM NaCl to remove the TCEP. A DBCO-PEG4-Malemide linker (Sigma-Aldrich cat#760676-5 mg) was resuspended at 5 mg/ml in DMSO. 2.5 mg of linker was added to 3 mg of protein in a 10 ml volume (final DMSO concentration was 5%). Linker was incubated with the reduced protein for 30 min at room temperature. An Ambicon 100 MW cutoff filter concentrator was used to remove excess linker by buffer exchange (Millipore Cat #UFC910096). Azide-PEG4-3M-012 (synthesized in house) was used for the final click chemistry step (Supplementary Fig. 8A). We added 0.5 mg of agonist to 0.5 mg of protein for final conjugation step and incubated at 37˚C for 6 h then 4 °C overnight. We removed excess agonist by buffer exchange using an Ambicon 100 MW cutoff filter concentrator. Conjugation efficiency was confirmed by mass spectrometry (Supplementary Fig. 8B).

### ELISA

The Ab response in mice was determined by endpoint titer ELISA. Briefly, 96-well plates were coated with 1 µg/ml of OspA-His of the determined serotype diluted in PBS and incubated overnight at 4 °C. The OspA-His was removed and the plates were blocked with 5% skim milk dissolved in PBST. After removing the blocking reagent, the primary serum samples were added after being serially diluted 2-fold in PBST starting at 1:400 dilution. The primary samples were added in equal volume to blocking solution for a final 50% blocking solution concentration. After a 1 h incubation with the primary antibodies, the plates were washed with PBST and incubated with Goat anti-mouse IgG (Invitrogen Cat# 32230) or Mouse anti-monkey, HRP-linked secondary Ab (Southern Biotech, 1:5000 dilution in blocking solution) for 1 h at room temperature. The secondary Ab was aspirated and washed and the plates were incubated with Sure Blue TMB peroxidase substrate (KPL, Gaithersburg, MD) followed by equal volume of stop solution (0.5 N sulfuric acid). Absorbance was measured at 450 nm. Endpoint titer was calculated in Graphpad PRISM version 7 with a threshold value that is approximately three times higher than background (0.2 AU).

### Statistics

*P*-values were calculated using Graphpad PRISM version 7 using a student *t*-test.

### Mass spectrometry

Conjugation of OspA-ferritin with 3M-012 was confirmed by mass spectroscopy. OspA-ferritin untreated or conjugated with 3M-012, were run on a Waters Acquity H-Class UPLC system with an in-line Acquity QDa detector. Proteins separated with a 30 min 0.1% formic acid in water to 0.1% formic acid in acetonitrile gradient run on an Acquity C18 column (Waters, Acquity UPLC BEH C18 1.7 µm #186002350). Intact masses were analyzed using Water MassLynx software with MaxEnt processing.

### Structural representations

Structural renderings of *H. plylori* ferritin (pdb 3BVE) and *B. burgdorferi* OspA (pdb 1OSP) were performed with the UCSF Chimera package, version 1.12 (http://www.cgl.ucsf.edu/chimera/). Chimera is developed by the Resource for Biocomputing, Visualization, and Informatics at the University of California, San Francisco (supported by NIGMS P41-GM103311).

### Animal ethics

All animal study experiments conducted in the U.S. were carried out in accordance with all federal regulations and were ethically approved by Sanofi Institutional Animal Care and Use Committee. All experimental animals in the *B. afzelii* challenge study were treated in accordance with the Animal Protection Law of the Czech Republic No. 246/1992 Sb., ethics approval No. 161/2011. The animal experimental protocol was approved by the Czech Academy of Sciences Animal Care & Use Committee (Protocol Permit Number: 102/2016). The rhesus monkey experiment were run in accordance with the recommendations of the Association for Assessment and Accreditation of Laboratory Animal Care International Standards and with the recommendations in the Guide for the Care and Use of Laboratory Animals of the United States—National Institutes of Health. The Institutional Animal Use and Care Committee of BIOQUAL approved these experiments IACUC protocol # 17-089 P. BIOQUAL’s animal facilities meet or exceed all AAALAC, USDA and OLAW standards for animal housing, and the surrounding environment.

### SDS-PAGE gels

All gels were derived from the same experiment and processed in parallel.

### Reporting summary

Further information on research design is available in the Nature Research Reporting Summary linked to this article.

## Supplementary information

supplemental-materials

reporting-summary

## Data Availability

The data that support the findings of this study are available from the corresponding author upon request.
