# Supplementary material for: Design of a broadly reactive Lyme disease vaccine
Source: NPJ Vaccines. 2020 May 1;5:33. doi: 10.1038/s41541-020-0183-8 (PMC7195412; doi:10.1038/s41541-020-0183-8)
Supplement: Supplementary file 1 — supplemental-materials [file 41541_2020_183_MOESM1_ESM.pdf]

Supplementary Figure Legends

Supplementary Figure 1. Dynamic light scattering of OspA-ferritin nanoparticles

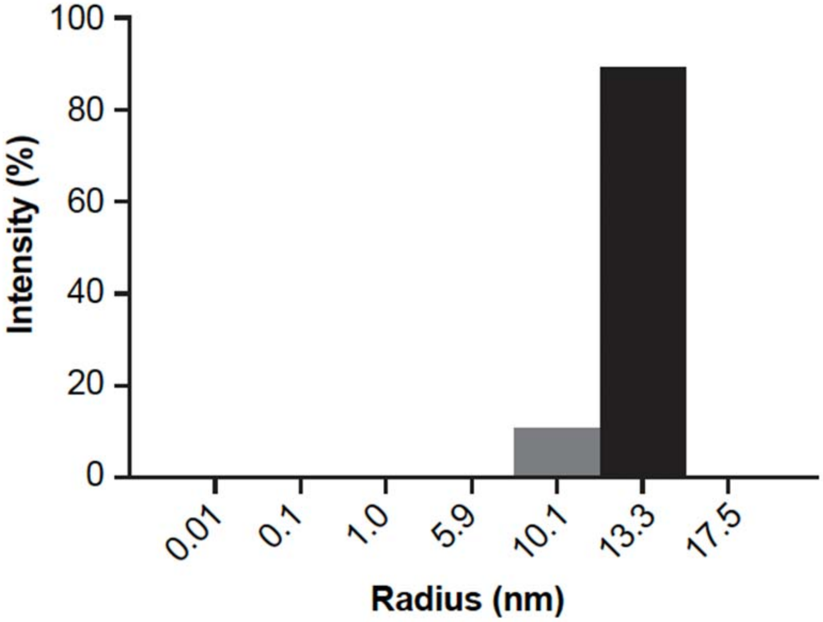

Dynamic light scattering profile of OspA-ferritin nanoparticles. Radius was 13 nm, 7.4% polydispersity and mass was 100%.

Supplementary Figure 2. Binding of LA-2 antibody to OspA-ferritin nanoparticles.

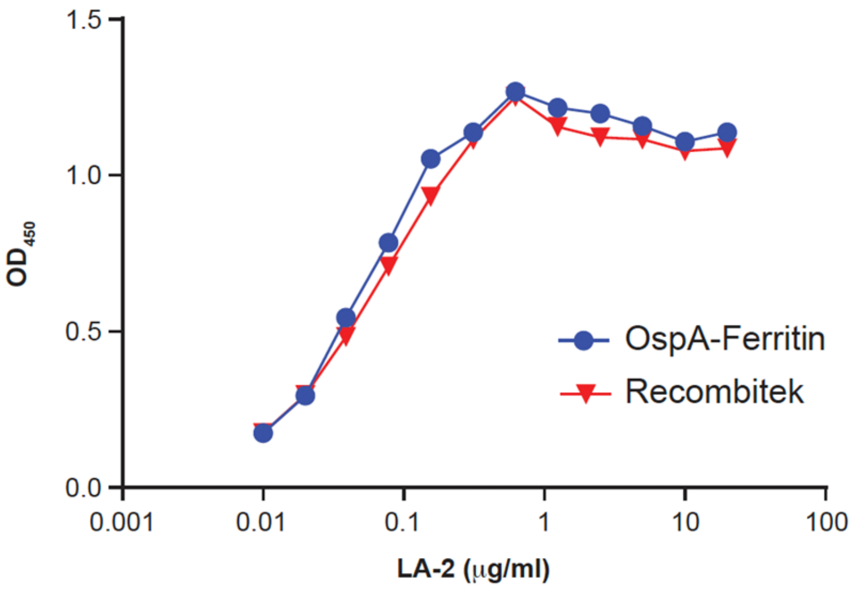

ELISA coated with either OspA-ferritin or Recombitek. Purified mouse LA-2 antibody was added in a dilution series to determine antibody binding.

Supplementary Figure 3. Immunogenicity and duration of the Ab response induced by serotype 1 OspA-ferritin nanoparticles compared to Recombitek^{TM}.

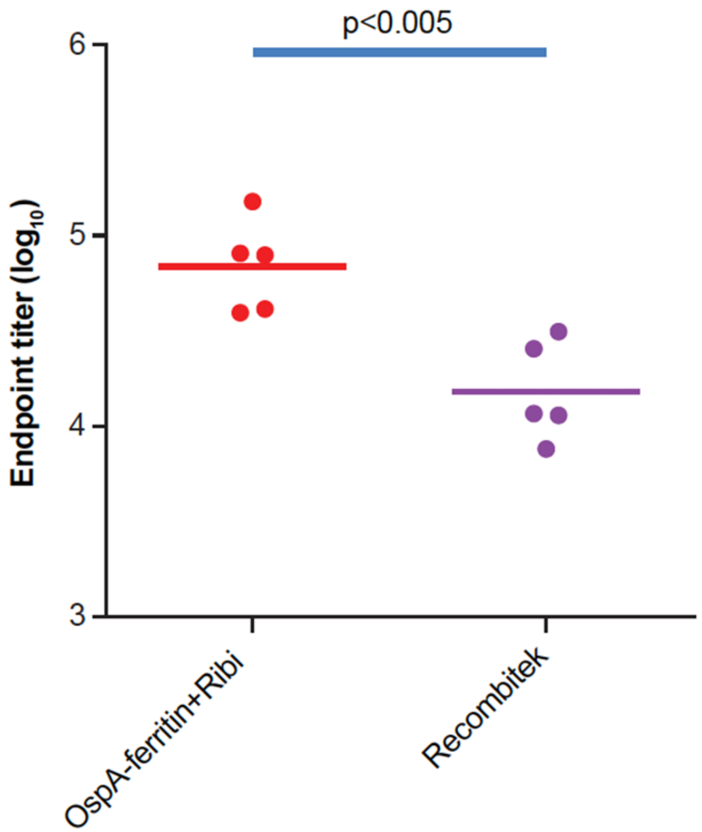

C3H mice (n=5) were immunized intramuscularly with the molar equivalent of 1 µg of the OspA-ferritin + Ribi adjuvant or Recombitek^{TM} at week 0 and week 4. ELISA titers were measured 21 wks after 2nd immunization (week 25) of each vaccine.

Supplementary Figure 4. Homology of LFA-1 to OspA serotypes

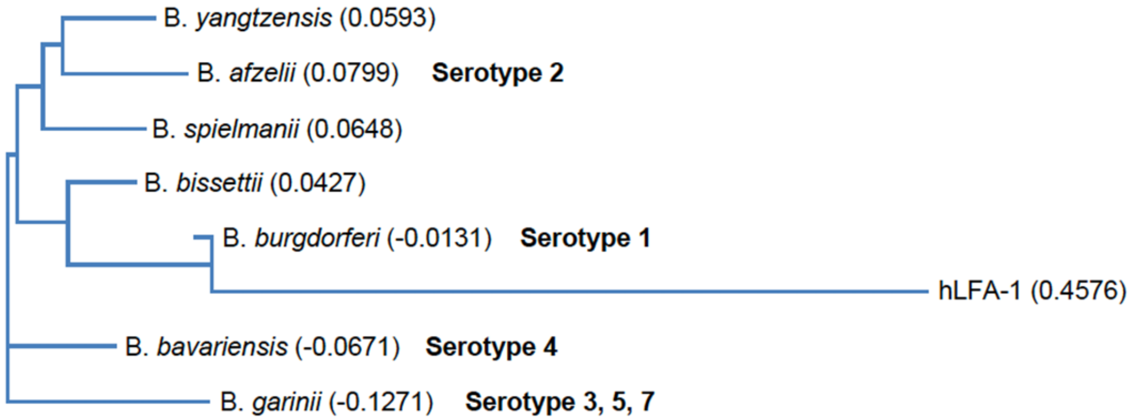

Phylogenetic tree showing the homology of OspA serotypes to human LFA-1 aa 326-173. The putative LFA-1 homology site in OspA serotype 1 is at aa165-173.

Supplementary Figure 5. Generation of alternative serotype OspA nanoparticles in *E. coli*.

Serotype	Strain
1	*Borrelia burgdorferi B31*
2	*Borrelia afzellii Pko*
3	*Borrelia garinii PBr*
4	*Borrelia bavariensis Pbi*
5	*Borrelia garinii Phei*
7	*Borrelia garinii T25*

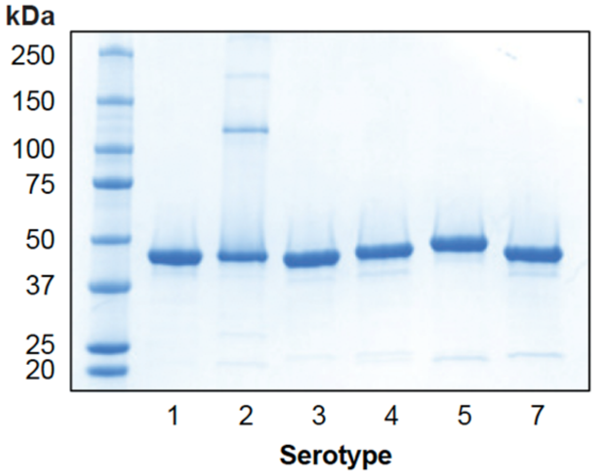

Biochemical analysis by SDS-PAGE of OspA-ferritin serotypes 1-5,and 7 purified by size exclusion chromatography.

Supplementary Figure 6. Body weight changes in mice after vaccination.

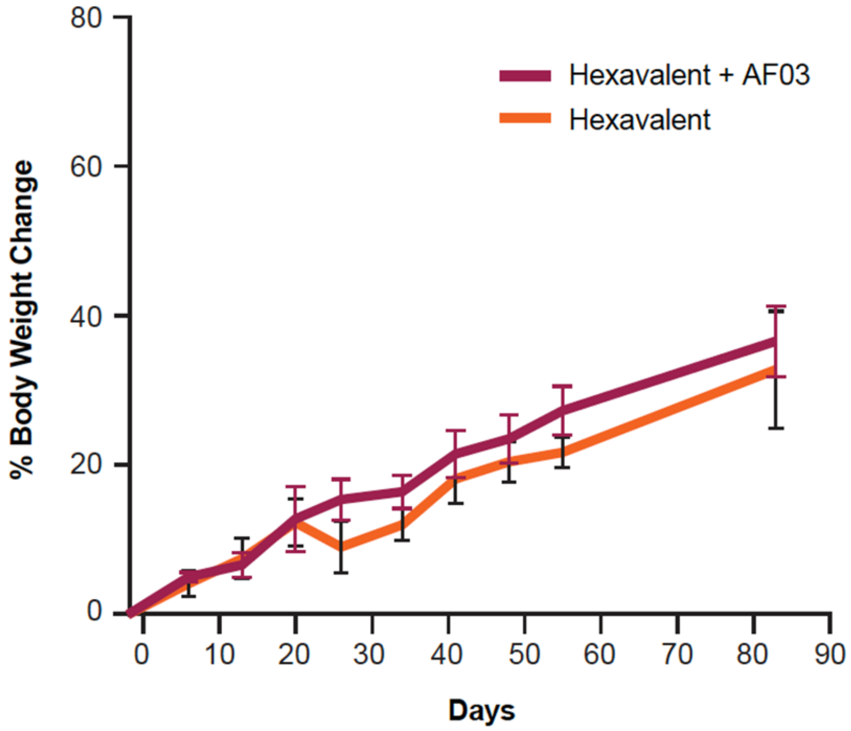

The % body weight change was calculated from the weight of the mouse at the first vaccination (day 0). C3H mice (n=5) were immunized intramuscularly at days 0 and 28 with 1 µg doses of the indicated vaccine. The hexavalent vaccine included OspA from serotypes 1,2,3,4,5,7 at the molar equivalent of 1 µg each. The error bars indicated the standard deviation of the mean.

Supplementary Figure 7. Purification and Immunogenicity of OspA-ferritin Serotype 6

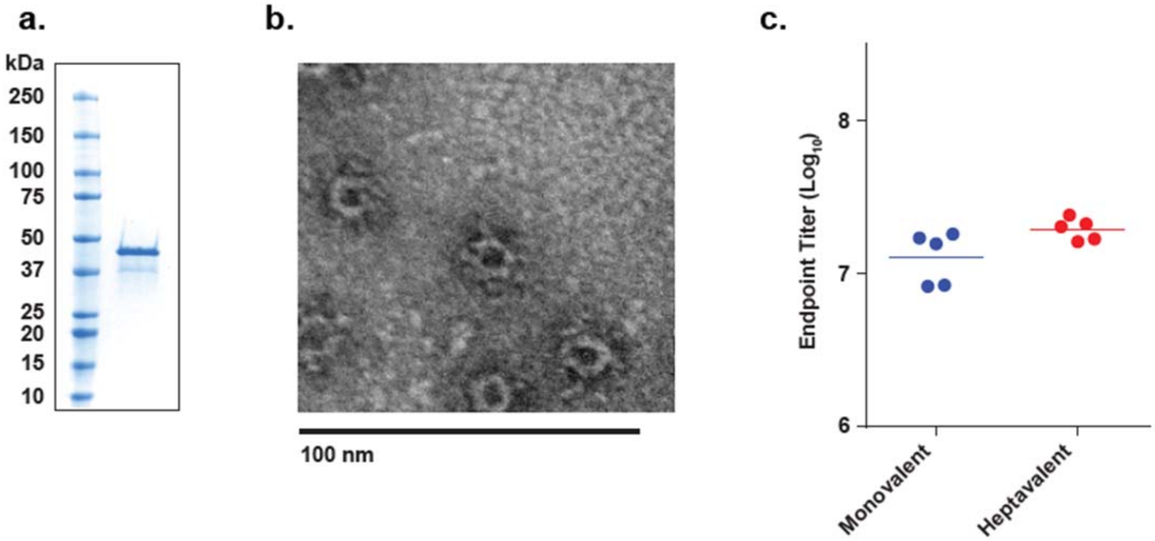

Biochemical analysis of OspA-ferritin Serotype 6 by SDS-PAGE after purification by size exclusion chromatography (A). Transmission electron microscopy image of OspA-ferritin serotype 6 nanoparticles (B). C3H mice (n=5) were immunized intramuscularly with the molar equivalent of 1 µg of the OspA-ferritin serotype 6 + Alum adjuvant (Monovalent) or OspA-ferritin serotypes 1-7 (Heptavalent) at week 0 and week 4. ELISA titers were measured 2 wks after 2nd immunization (week 6) of each vaccine. ELISA plate is coated with OspA-His Serotype 6.

Supplementary Figure 8. Conjugation of OspA-ferritin nanoparticles to TLR 7/8 agonist 3M-012.

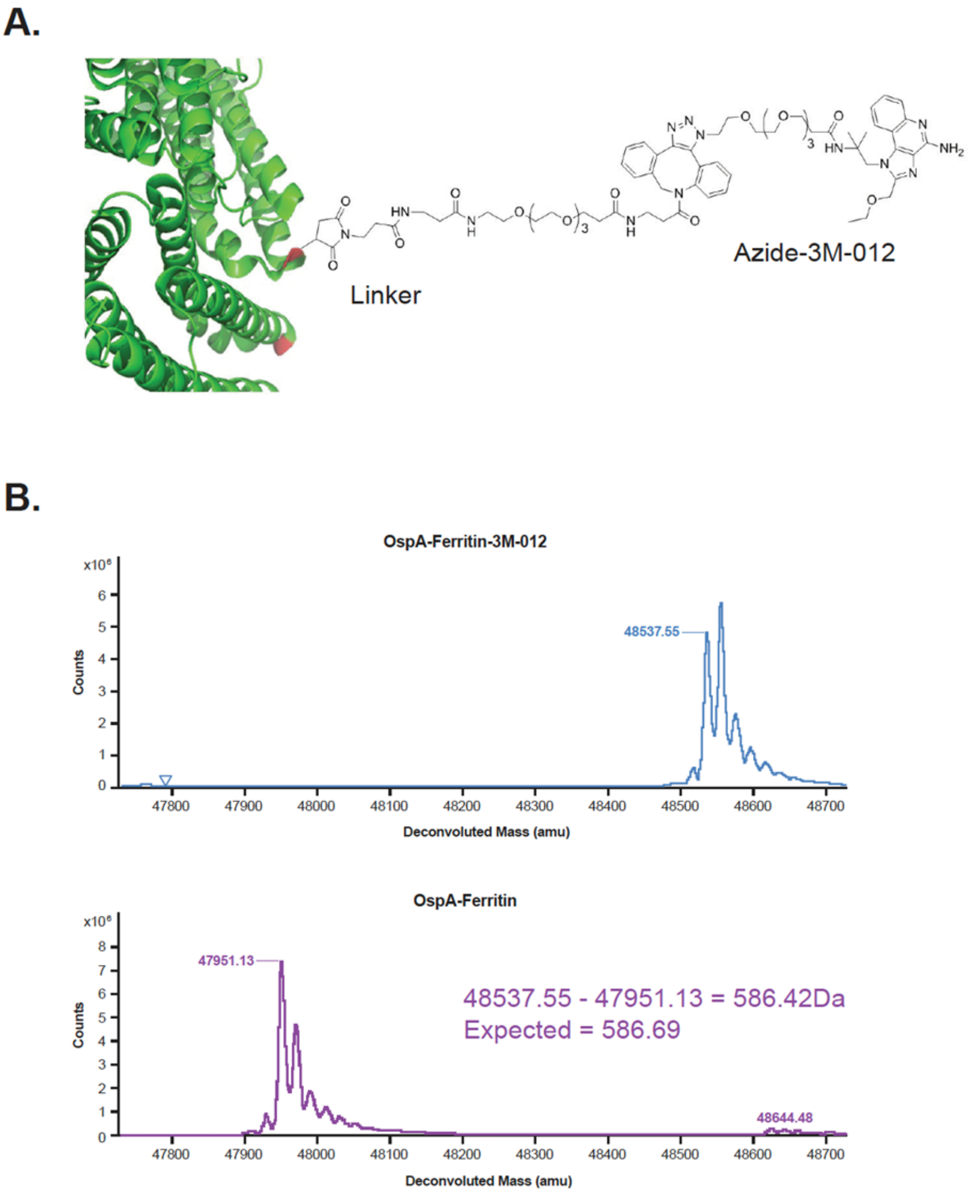

A 2 step click chemistry strategy was used to attach 3M-012 to the ferritin nanoparticle. A DBCO-Peg4-maleimide linker was first attached to the cysteine on ferritin. After excess linker was removed, the Azide-3M-012 (A) was added. Mass spec analysis showing the unconjugated and conjugated protein with a Mass shift of 586.7 Daltons consistent with the addition of 3M-012 (B).
